# Conserving Biogeography: Habitat Loss and Vicariant Patterns in Endemic Squamates of the Cerrado Hotspot

**DOI:** 10.1371/journal.pone.0133995

**Published:** 2015-08-07

**Authors:** Pietro L. H. de Mello, Ricardo B. Machado, Cristiano de C. Nogueira

**Affiliations:** 1 Programa de Pós-Graduação em Ecologia, Instituto de Ciências Biológicas (IB), Universidade de Brasília (UnB), 70910–900, Brasília, DF, Brazil; 2 Departamento de Zoologia, Instituto de Ciências Biológicas (IB), Universidade de Brasília (UnB), Brasília, DF, Brazil; Università degli Studi di Napoli Federico II, ITALY

## Abstract

Little is known about the threat levels and impacts of habitat loss over the Cerrado Squamate fauna. The region is under severe habitat loss due to mechanized agriculture, accelerated by changes in the Brazilian National Forest Code. The Squamate fauna of the Cerrado is rich in endemics and is intrinsically associated with its surrounding microhabitats, which make up a mosaic of phitophysiognomies throughout the region. Herein we evaluate current conservation status of Squamate biogeographic patterns in the Brazilian Cerrado, the single savanna among global biodiversity hotspots. To do so, we first updated point locality data on 49 endemic Squamates pertaining to seven non-random clusters of species ranges in the Cerrado. Each cluster was assumed to be representative of different biogeographic regions, holding its own set of species, herein mapped according to their extent of occurrence (EOO). We then contrasted these data in four different scenarios, according to the presence or absence of habitat loss and the presence or absence of the current protected area (PA) cover. We searched for non-random patterns of habitat loss and PA coverage among these biogeographic regions throughout the Cerrado. Finally, with the species EOO as biodiversity layers, we used Zonation to discuss contemporary PA distribution, as well as to highlight current priority areas for conservation within the Cerrado. We ran Zonation under all four conservation scenarios mentioned above. We observed that habitat loss and PA coverage significantly differed between biogeographic regions. The southernmost biogeographic region is the least protected and the most impacted, with priority areas highly scattered in small, disjunct fragments. The northernmost biogeographic region (Tocantins-Serra Geral) is the most protected and least impacted, showing extensive priority areas in all Zonation scenarios. Therefore, current and past deforestation trends are severely threatening biogeographic patterns in the Cerrado. Moreover, PA distribution is spatially biased, and does not represent biogeographic divisions of the Cerrado. Consequently, we show that biogeographic patterns and processes are being erased at an accelerated pace, reinforcing the urgent need to create new reserves and to avoid the loss of the last remaining fragments of once continuous biogeographic regions. These actions are fundamental and urgent for conserving biogeographic and evolutionary information in this highly imperiled savanna hotspot.

## Introduction

Biodiversity and anthropogenic threats are not randomly distributed throughout the world [1–3]. As a response to intense habitat losses and the worldwide biodiversity crisis [4–8] a new branch of biodiversity science gained strength in the 21^st^ century: Conservation Biogeography [2,3]. This new area applies biogeographic principles, theories, and analyses [9], to provide solutions to the urgent problems related to the conservation of biodiversity [2]. Diverse methods to optimize the distribution of priority areas for biodiversity have already been suggested [2], including the hotspot approach [1]. This approach incorporates habitat loss and species endemism to map regions of high conservation importance [1], an idea which gained strength in the early 2000s, mostly due to the recent alarming rates of global habitat loss [10,11].

Endemism patterns and their derived biogeographic units are central concepts in biogeography [12], providing important information on which spatial portions of biodiversity should be conserved [2,13]. However, conservation planning initiatives often neglect biogeographic and endemism patterns [14]. Currently, diverse and highly threatened regions such as the Brazilian Cerrado hotspot are still poorly studied concerning the detection and conservation of their biogeographic patterns and processes [15]. Nevertheless, a recent study using Biotic Element Analysis detected significant levels of regionalization for the Cerrado Endemic Squamate fauna, recovering seven distinct biogeographic units, through Biotic Element (BE) analysis, based on 49 endemic Squamate species [16].

The Biotic Element (BE) analysis is a relatively recent method of biogeographic pattern detection that tests central assumptions of the vicariant diversification model [17,18], one of the main processes to affect the distribution of biodiversity throughout the planet [13,19,20]. According to BE analysis, if vicariant processes were important in the past, significantly co-distributed groups of species (Biotic Elements, see [18]) must exist and be detectable, as signatures of historical processes of biotic regionalization [17,18]. In addition, phylogenetically close species must compose distinct BEs, because of historical allopatry [17,18]. Thus, BE can be interpreted not only as a mere spatial pattern, but also as a consequence of historical processes of vicariant speciation, caused by the appearance of historical biogeographic barriers [17].

The Brazilian Cerrado is a region with a particularly complex and dynamic geomorphological history [17,21,22]. In addition, the Cerrado, due to its high levels of vascular plant endemism [23,24] and high rates of habitat loss [25], it is the single savanna among the 34 global biodiversity hotspots [1,26]. Its biodiversity and associated threats are not distributed at random, with deforestation following a south-north trend [15,25,27]. Most habitat loss has ocurred in open, interfluvial flatland savanna habitats [28] in the southern portion of the region.

The Cerrado harbors a rich (over 260 species) and highly endemic Squamate fauna, that contains at least 103 endemic species (about 40% endemism), showing significantly regionalized ranges [16]. Endemism patterns for different Cerrado taxa have already been described (e.g. [16,21,29]), however, spatially explicit data on Cerrado biogeographic units have never been directly used in conservation planning analyses, which remain scarce in the Cerrado (but see [30–33]). Moreover, the rich and highly endemic Cerrado Squamate fauna is dominated by species tightly associated to specific microhabitats [34,35] unevenly distributed in habitat mosaics. The observed regionalized, significant patterns of species co-occurrence in the group agree with the predictions of the vicariant model of diversification, indicating that current diversity and distributional patterns are a possible result of a long history of allopatric diversification and *in situ* speciation [16], enhancing the importance of biogeographic patterns as a surrogate for historical processes acting to shape current biodiversity patterns [16,17,21].

Even with recent efforts to expand the coverage of threat assessments in Reptilia [8] only twelve of the 103 Cerrado endemic Squamate species [16] have been assessed in the IUCN Red List until January 2015 [36]. Additionally, it is expected that habitat loss, the single most important threat to reptiles worldwide [7,8,37,38], will increase in Brazil after the recent approval of the new National Forest Code [39], bringing harmful consequences not only for reptiles [40] but to all vertebrate taxa [41–44]. This new Brazilian National Forest Code removed the protection of mountaintops, formerly considered as Areas of Permanent Protection (areas that must be permanently protected, even if outside formal reserves), and reduced the minimum protected width of gallery forests and riparian habitats [39]. Most importantly in a biogeographical context, the new code opened the possibility of environmental compensation in different areas of the same biome: thus, one can now extensively deforest one region and compensate the damage by sparing the natural vegetation in a different region in the same biome. The result is that different portions of the Cerrado will now tend to show very different levels of deforestation, and impacts will no longer be compensated at the small, local, scale (as required in the formed version of the code, with micro-watersheds as basic units), but in a much wider, continental scale.

These modifications in Brazilian environmental laws will probably enhance the threats to Cerrado biodiversity, especially in its open interfluvial plateaus, highly prone to mechanized agriculture [26, 27]. Due to the high microhabitat specificity of Cerrado Squamates [34,35], the severe impacts of habitat loss threatens to erase ancient and highly complex evolutionary patterns and processes [16]. Consequently there is a clear need for a first approach with systematic conservation planning [45], for Cerrado endemic squamates.

Therefore, the first aim of our study was to test the presence of non-random differences in habitat loss and protected area distribution among different biogeographic units of the Cerrado, i.e. to evaluate if biogeographic patterns the and processes are well represented in conservation strategies in the Cerrado. Secondarily, using a conservation planning approach, we assessed the influence of habitat loss and current federal PA distribution in conservation prioritization scenarios for the Cerrado, providing a map of potential of high conservation priority areas according to Cerrado biogeographical patterns.

## Materials and Methods

### Study area

The Cerrado is the second largest South American phytogeographical domain [15,24,46] covering ca. 2.03 million km^2^, or approximately 23% of the Brazilian territory. It is a seasonally dry tropical savanna [47], with two major geomorphological units [21,22]: a) gently rolling or level headwater plateaus, dominated by open grassy savannas and grasslands, and b) peripheral depressions, that harbors a more complex matrix of savannas and semi deciduous forests, crossed by widespread tracts of gallery forests along major drainage systems [23,48]. Detailed data on Cerrado ecology and natural history can be found elsewhere [49].

### Data sources

We obtained a total of 451 point localities for 49 endemic Squamates belonging to Endemic Biotic Elements (BE) detected in a previous biogeographic analysis [16]. Species occurrence records derive from a revised database of vouchered point locality records in zoological collections and in a compilation of more recent literature records, from 2010 onwards, complementing the database used in [16] (S1 Table). The georreferenced information for each vouchered specimen was recovered using the information associated to vouchered specimens, or from the localities provided in taxonomical studies. All data were taken from sources from the beginning of the 20th century onwards.

Prior to building each species distribution, we used the official map of Brazilian biomes [50] to define approximate limits of the Brazilian Cerrado. We restricted all projections of land cover changes and species ranges to these boundaries. We obtained land coverage modifications for the Cerrado (2002 and 2010) from the Project of Satellite Deforestation Monitoring of Brazilian Biomes (*Programa de Monitoramento dos Biomas Brasileiros—PMDBBS*) [51]. We used IUCN categories I-IV in [52] to define strictly protection areas in the current Brazilian protected area (PA) system [53]. To maintain consistency we represented all variables at 10 x 10 km spatial resolution, and processed species distribution data and habitat loss in a geographical information system. All georeferencing and mapping procedures were developed in ArcMap 10.2.2 [54].

### Estimating species extent of occurrence and biotic element range

We determined the extent of occurrence (EOO) [55,56] for each species pertaining to each one of the seven BE identified in [16], as well as its respective habitat loss and PA coverage. We calculated each species range as extent of occurrence (EOO) [55,56]. The EOO is defined as “the area contained within the shortest continuous imaginary boundary which can be drawn to encompass all the known, inferred or projected sites of present occurrence of a taxon, excluding cases of vagrancy” [55,56]. Following the IUCN guidelines [55,56], for species with 3 or more different localities (see S1 Table) we built each species extent of occurrence using the MCP approach. This approach consists in the smallest polygon in which no internal angle exceeds 180° degrees and which contains all the sites of occurrence [55,56]. When a species had only one or two different localities we built a buffer with a 10km radius around its referenced capture point (for a similar approach, see [57]). Although methods such as MCP have received considerable attention, resulting in positive [55] and negative [58] conceptions about their utilization, we stress that we are not looking for fine scale, refined species distribution data. Instead, we investigate macroscale spatial patterns of habitat loss and PA coverage of the biogeographic regions across the Cerrado region. Thus, simply mapping ranges as EOO for each species following IUCN guidelines [54,55], instead of species distribution models [59], is sufficient for our analytical needs.

**Fig 1:**
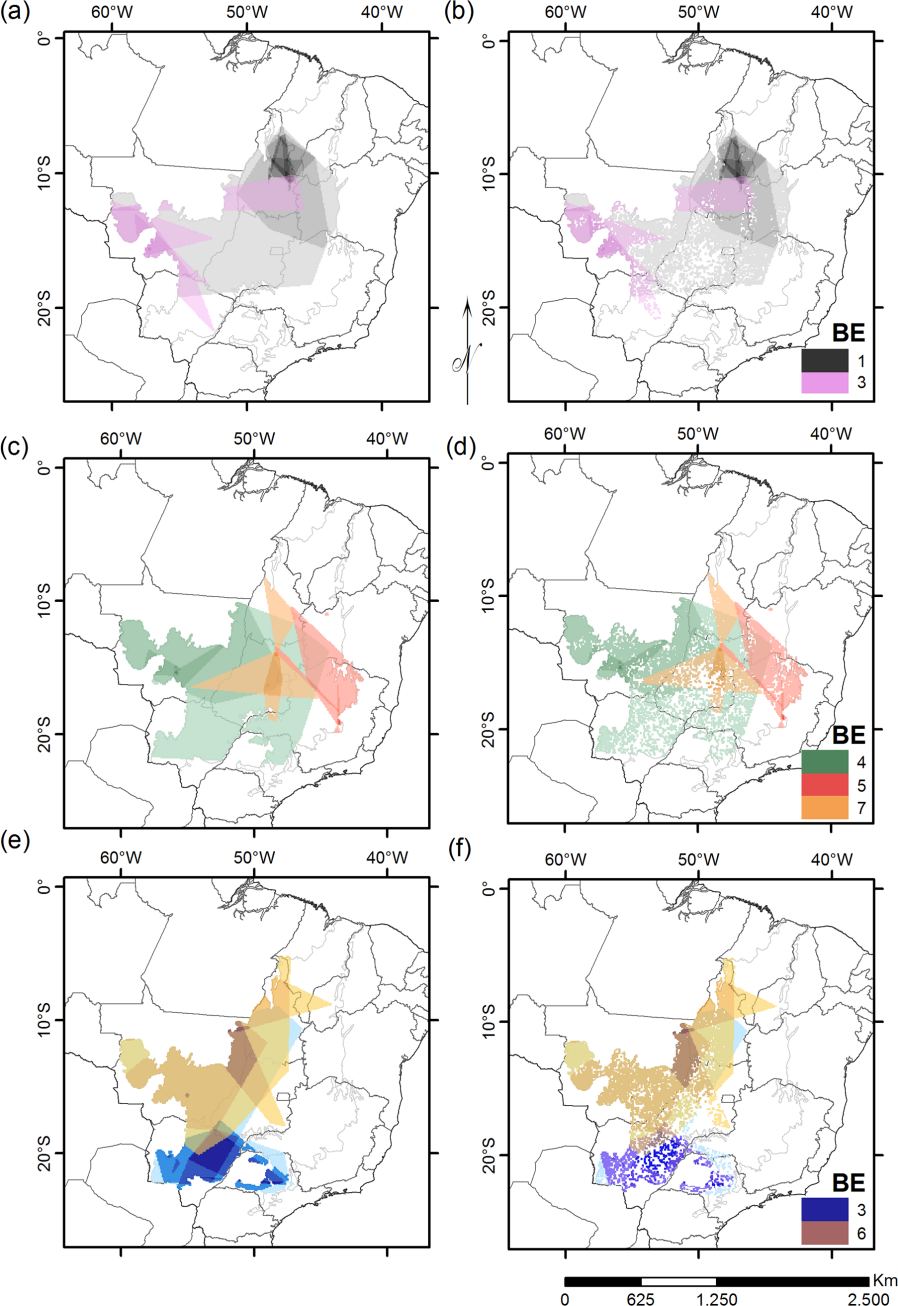
Biotic Elements extent under original and current natural vegetation cover. Biotic Elements (BE) distribution under the Cerrado vegetation original extent (left), and considering current levels of habitat loss (right) (until 2010). Each BE is represented by shades of a single color. The darker the shade, the more projected species distributions for that BE are overlapping at that area. Shades of black: Tocantins-Serra Geral BE I (a and b). Shades of purple: Paraguay-Guaporé BE II (a and b). Shades of blue: Paraná-Paraguay BE III (c and d). Shades of Green: Guimarães-Roncador BE IV (c and d). Shades of red: Espinhaço BE V (e and f). Shades of yellow: Araguaia BE VI (e and f). Shades of orange: Central Plateau BE VII (e and f).

Since every BE has its own set of species, we built the BE ranges by merging its species distributions (Fig 1). We assumed that BE ranges serve as area surrogates of the different biogeographic units. Therefore, detected and quantified patterns of anthropic influences over each BE, such as deforestation or PA coverage, are assumed to be also happening at the level of the biogeographic region each BE represents. We built two sets of EOO for each species: the original EOO, which did not account for habitat loss; and the current EOO, which takes into account only the remaining natural vegetation cover within the species ranges. To obtain the species current EOO we clipped each species original EOO with a land coverage modifications map provided by the PMDBBS [51] (Fig 1D, 1E and 1F). For calculating original and current habitat loss within BEs, we merged all ranges from a BE into a single shape file. This was done in order to avoid counting the same area twice when different species distributions overlapped.

### Biogeographic patterns and habitat loss

We analyzed the conservation status of the biogeographic regions by comparing species habitat loss and PA coverage both within and among biotic elements (BEs) [17]. The seven proposed BEs (see [16]), located in different portions of the Cerrado (Fig 1), are: 1) Tocantins-Serra Geral; 2) Paraguay-Guaporé; 3) Paraná-Paraguay; 4) Guimarães-Roncador; 5) Espinhaço; 6) Araguaia; 7) Central Plateau. Due to differences in area between biogeographic units, we chose to compare the proportion of habitat loss among BEs, instead of absolute values.

We calculated the expected habitat loss for each species as the proportion of each species original EOO, under the average habitat loss of all species belonging to its BE, as in previous studies [60]. Observed and expected habitat loss values were then *logit* transformed [61] for all analysis in R’s package *car* [62]. To calculate differences between expected and observed habitat loss for each species within each BE we used Kolmogorov-Smirnoff tests (see [63]). We compared the differences between observed habitat loss, and PA coverage among different BEs using Kruskal-Wallis tests [64] and multiple comparison tests (see [65]), in package *pgirmess* in R [62].

### Systematic conservation planning

We used Zonation [66,67] software in order to maximize conservation benefits through a systematic conservation approach. Zonation is one of the most widely used decision–support tools in conservation planning [68]. The algorithm generates a hierarchy of priority areas [66], by iteratively removing all cells in a landscape, using a marginal loss criterion to decide which cell is removed after each step (see [69]). In our setup we chose to run Zonation v. 4.0 with core-area zonation, to prioritize areas with unique species records [70]. Zonation demands a set of biodiversity features as basic input (see [70]), such as species extent of occurrence, in raster format. We used the Core-Area Zonation (CAZ) [71] as our cell-removal rule. This method aims to minimize biological loss across the study area.

The Zonation analysis works as follows: as a consequence of the overlay of the distinct biodiversity features, each pixel receives a set of values. These values are independent among them, and refer to each species distribution that occurs on that pixel. The algorithm picks as the next cell to be excluded, the cell that has the smallest value of the most valuable occurrence. The most valuable occurrence is the highest value of occurrence of any species in the pixel, and varies as the algorithm ‘runs over’ the input maps. It always changes because the program basically recalculates the proportion of the remaining distribution of every species in the set of the remaining cells every time any cell is withdrawn. Thus, when a part of the distribution of a species is removed, the value of the remaining distribution areas located in each remaining cell (its value of occurrence) goes up, i.e. the value of this species in all of its remaining pixels increases. CAZ, therefore, tries to retain core areas of all species until the end of cell removal [70].

It is advisable [70] to calibrate a set of different Zonation parameters heuristically before running final models. Therefore, to determine whether or not to add edge points in Zonation, we made a set of preliminary runs comparing the resultant outputs. The standard Zonation configuration does not apply the ‘add edge points’ parameter and, therefore, Zonation runs over the input biodiversity features contiguously, starting from the borders [70] and moving onwards from this point. As for boundary penalty method (BLP), we chose the boundary length penalty [70]. The BLP value is a penalty associated with the extent of a pixel which is ‘exposed’ to ‘non habitat’ areas, e.g. areas that have already been withdrawn from the landscape by the program. Therefore, as the BLP value—which is manually provided—gets higher, so does the penalty of having boundaries with removed pixels. After preliminary tests, we chose the boundary length penalty (BLP) of 0.001, that empirically avoided pixel aggregations [71].

From this preliminary calibration procedure, we used a warp factor of 1 and a boundary length penalty of 0.01, for all runs, indicated in Zonation user`s manual [70]. The warp factor [70] defines how many cells are removed at a time; therefore, we chose to remove one pixel at a time, which results in a more refined solution. When defining which parameters to run the program, we chose to use the Zonation feature to add edge points [70] throughout the Cerrado. We thus added hypothetical borders to the landscape, so that the program could perceive islands of poor habitat margined by “shores” of good habitat, without risking valuable cells [70]. This allowed Zonation to highlight the very top pixels for conservation, irrespective of fragmentation or current PA coverage. This was considered to be the best alternative to our ambitions since we are looking to conserve biogeographic patterns, i.e. we are looking to retain as much of original ranges as possible, not only the pixels with the highest overall richness. When testing hypothesis (iii) and (iv) we added current protected area (IUCN categories I-IV) distribution as a mask layer.

To investigate the effects of habitat loss and protection scenario on Cerrado conservation priority areas, we ran Zonation software under four scenarios: (a) no habitat loss (i.e. original potential ranges), and no PA coverage; (b) no habitat loss, and current PA coverage (strict protection areas IUCN categories I-IV as permanently PA in the analysis mask layer); (c) habitat loss until 2010, and no PA coverage; and (d) habitat loss until 2010, and current PA coverage. We chose to run Zonation both with and without habitat loss to look for differences among potential complementary regions for current protection area distribution (i.e. with current PA coverage) [72,73], and areas that would be prioritized when not taking in account such current protection areas [74]. We presented all resulting maps emphasizing the top 25% priority areas, and a map with the original values for the scenario with current PAs and habitat loss is provided in S1 Fig.

## Results

### Biogeographic patterns and habitat loss

**Fig 2:**
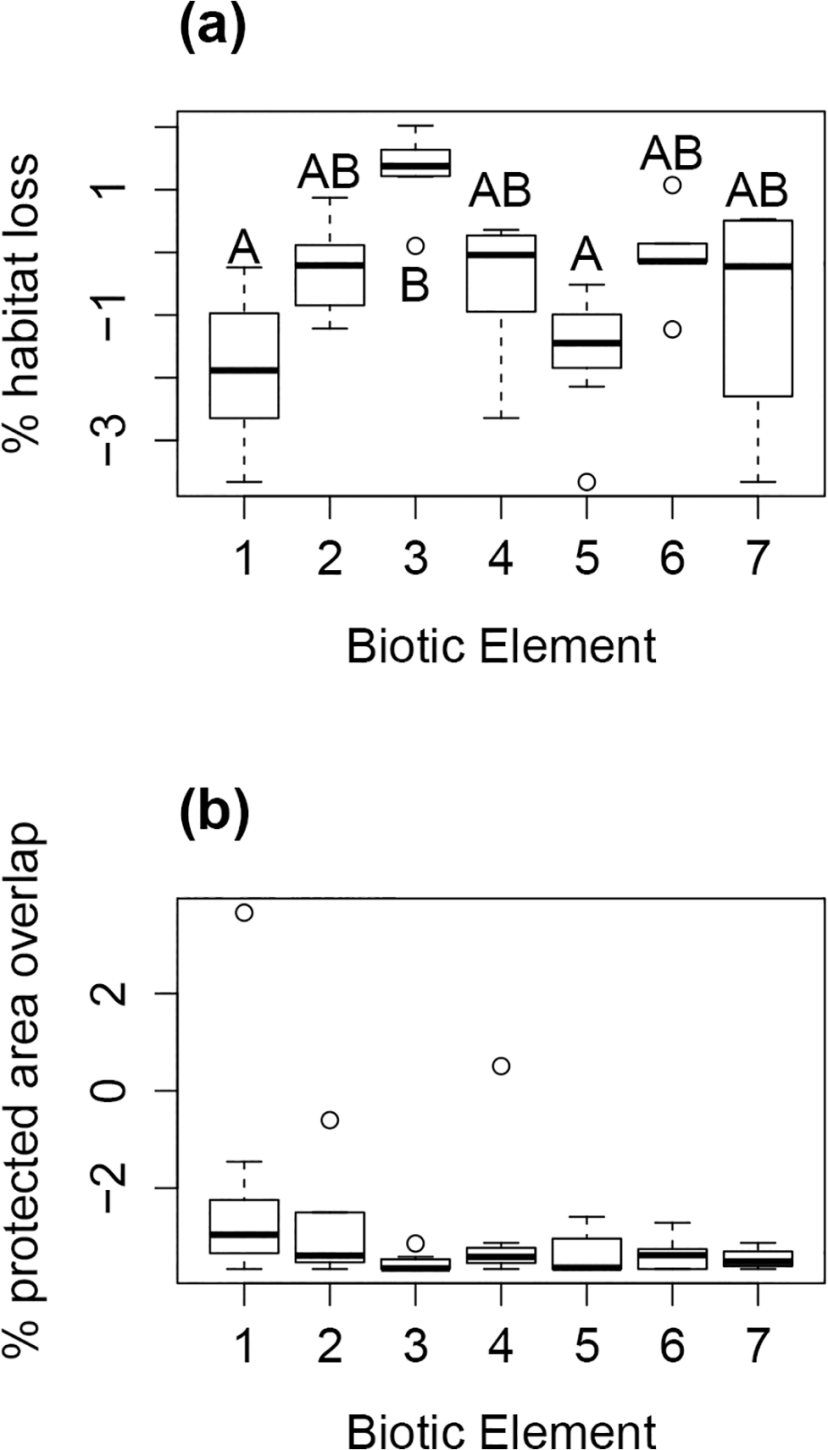
Habitat loss and protected area (PA) cover per Biotic Element (BE). Boxplots indicating: (a) The recorded percentage of habitat loss, per BE (1–7) until 2010; (b) The percentage of overlap of each BE natural cover with Cerrado federal PAs e. Horizontal bars = median; box = first and third quartiles; whiskers = minimum and maximum values. Percentages in the Y axes are logit transformed. Biotic elements sharing common letters (A or B) had non-significant differences.

We estimated ranges for all 49 endemic Squamate species forming biotic elements (see Fig 1). We found no significant differences between observed and expected habitat loss among species within each BE (S1 Table). Current habitat loss, however, was significantly different among BEs (Kruskall-Wallis = 28.1858, df = 6, *P* <0.005), with losses in BE 3 (Paraná-Paraguay) significantly higher than those in BE 1 (Tocantins-Serra Geral, obs. dif. = 31.28, critical dif. = 19.76) and BE 5 (Espinhaço, obs. dif = 29.30, critical dif = 22.01) (Fig 2A, see also Fig b and d).

### Protected area cover and systematic conservation planning

**Fig 3:**
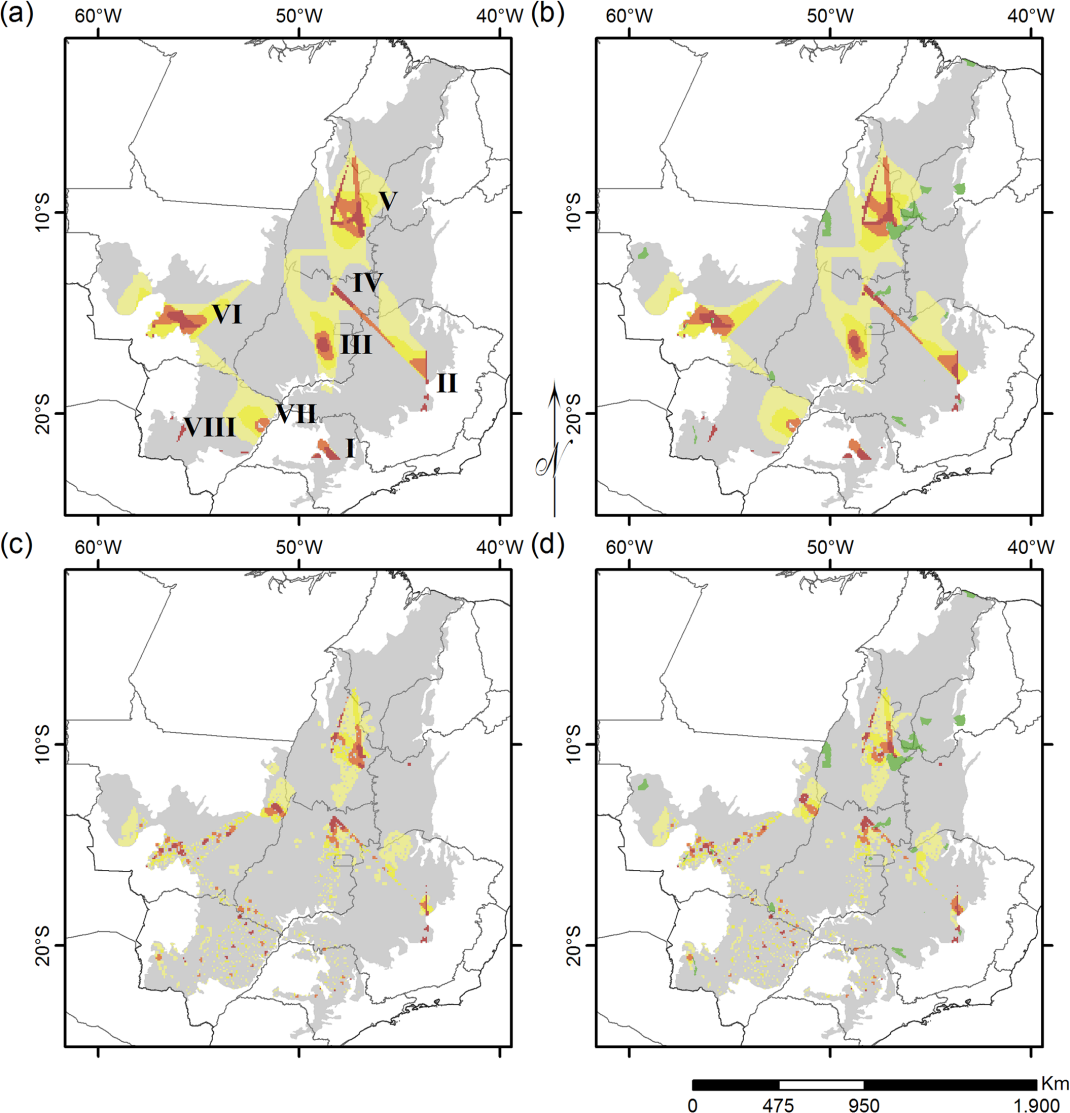
Priority regions for Cerrado squamate conservation. High priority regions for conserving endemic Squamates in the Cerrado, under four scenarios: (a) without habitat loss and protected area coverage; (b) without habitat loss but including current protected area coverage; (c) considering habitat loss, but without protected area coverage (d) considering both habitat loss and current protected area coverage. Numbered priority regions in letter (a): I–São Paulo; II–Serra do Cipó; III–Brasília; IV–Chapada dos Veadeiros; V—Serra Geral do Tocantins; VI–Chapada dos Guimarães; VII–Três Lagoas; VIII–Serra da Bodoquena. Gradient colors are as follows: dark red, the best 2% of the landscape; orange, the best 2–5%; yellow, the best 5–10%; light yellow, the best 10–25%. Protected areas (IUCN categories I-IV) are represented in dark green.

In general, species in BEs were poorly covered by protected areas (PA), with an average of 2% PA coverage (Fig 2; S2 Table). We could not detect significant differences of PA coverage among BEs (Kruskall-Wallis = 7.6359, df = 6, *P* = 0.266). Priority areas detected with Zonation were generally similar across all scenarios (Fig 3). There are basically eight different regions pointed out as priorities, represented by different numbers in Fig 3 and named after nearby PA, if there is any. For all scenarios, larger priority area clusters are located in the northern portions of the Cerrado (Fig 3), overlapping the range of species in BE 1 (Tocantins-Serra Geral, shown with brown borders, see Fig 3). Analyzing each scenario separately, when habitat loss is not considered (Fig 3A and 3B), priority areas are segregated in small patches throughout the Cerrado. However, when habitat loss is accounted for (Fig 3C and 3D), priority areas in the southern and southwestern portions of the Cerrado become highly scattered in small, disjunct fragments. These highly impacted priority areas overlap with ranges from species in BE 3 (Paraná-Paraguay, shown with red borders, see Fig 3).

Current PAs were partially embedded in priority areas (see Fig 3B and 3D), but only priority region V (Serra Geral do Tocantins) (Fig 3A) is significantly represented by the current PA network. Larger PAs are found mainly at the northern portion of the Cerrado region, coinciding with priority areas within the Tocantins-Serra Geral BE range (outlined with brown borders, see Fig 3B and 3D). However, there is a clear lack protection in the south-southwestern portion of the Cerrado, regions I (São Paulo), VI (Chapada dos Guimarães) and VIII (Serra da Bodoquena), which overlap with ranges of the highly deforested BE 3 (Paraná-Paraguay). The entire extension of this southern Cerrado region is protected by only three very scattered relatively large PAs: Emas National Park located between regions VI and VII, Serra da Bodoquena National Park, west of priority region VIII, and Serra da Canastra National Park, located between priority regions I and II.

## Discussion

### Current protection of endemic Cerrado Squamates

Conservation biogeography should provide solutions that incorporate different conservation scenarios (cf. [75,76]). Thus, when we built two scenarios with PAs as mask layers, and two scenarios without mask layers, we expected to obtain different sets of potential priority areas for conservation. As previously stated, we expected that if important areas were not represented in current PA distribution, Zonation would point these areas as conservation priorities. On the other hand, if such important areas were part of the current protected areas distribution, then different areas would be signaled as conservation targets.

Contrary to our assumption that PAs would bias the representativeness of the original regional species diversity and distribution data set [70], they had no significant influence on the prioritization results in our study (Fig 3). Furthermore, Zonation did not seem to cluster priority areas around current protection areas, in scenarios ‘b’ and ‘d’ (see Fig 3C and 3D). It seems that, in general, current PA coverage would only influence the priority area location output from Zonation either when its coverage is significantly large relative to the whole region extent, e.g. fully covering at least one of the priority areas highlighted when considering the species original distribution; or if they were to represent an area with a particular set of biodiversity features, e.g. a gathering of restrict endemic species. Both scenarios could, consequently, raise the value of occurrence of a once secondary biodiversity feature, signaling it as a priority area for conservation when current protected area coverage is considered. Clearly, this is not the case for the Cerrado, whose realtively small protected area network, covering less than 2% of the region [28], was not large enough to significantly alter the distribution of spatial priorities at the biome scale.

Despite their small overall area, our results show that current protected areas partially overlap small fractions of priority areas II (Espinhaço), IV (Chapada dos Veadeiros), V (Serra Geral do Tocantins) and VI (Chapada dos Guimarães) (Fig 3A), highlighting the importance of the current PA in safeguarding Cerrado species diversity and biogeographical patterns. The clear overlap between the top priority areas in the nucleus of region VI and Chapada dos Guimarães National Park suggests that the existence of the park could be granting the preservation of historical biogeographic patterns in the region, even though its creation in 1989 [77] was not based in standard systematic approach of conservation as defined in the conservation planning literature [45]. Therefore, this situation stresses that although sometimes we may not truly know if we are conserving what we say we are [78], it is important to preserve and adequately maintain the reserves already established [79], because they are an effective in reducing deforestation and representing species regional diversity [80]. In fact, many biogeographical areas now largely affected by habitat loss, such as the entire Paraná-Paraguay BE in southern portion of the domain, have their last remnants within Cerrado protected areas (Emas, Serra da Canastra and Serra da Bodoquena National Parks). If not for these key areas, the entire biotic element would now be almost totally converted to anthropic areas.

### Threatened biogeographic patterns

Overall, irrespectively of the region, every species suffered decreases in current ranges as an effect of habitat loss (see S1 Table), a consequence of the massive and widespread habitat losses driven by the use of mechanized agriculture [27,28]. Nevertheless, we obtained significant differences in habitat losses between BE III (Paraná-Paraguay) and BEI (Tocantins Serra-Geral), and BE III and BE IV (Espinhaço) (Fig 1). It is noteworthy that the most imperiled BE (BE III—Paraná-Paraguay) is located at the southern portion of the Cerrado, which overlaps with Brazil’s most populated and developed region, that holds the three largest metropolitan regions in the country [81]. On the other hand, the least imperiled BE (BE III—Tocantins-Serra-Geral), i.e. the BE which held least habitat losses and proportionally most of its species EOO under PA areas, is located at the Northern portion of the Cerrado, distant from the same large Brazilian economical centers [82], and showing least developed and less populated cities than their southern counterparts [81,82].

Biotic element IV, the other biographical unit suffering significantly smaller losses than BE III, is the Espinhaço region. Even though the Espinhaço (BE IV) is geographically close to the massive economical centers in the south-eastern region of Brazil [81,82] (see Fig 1), it is a region formed mainly by steep and rocky outcrops [46], which potentially hampers any attempt of implementation of mechanized agriculture, or anthropic occupation, the main drivers of deforestation not only in Brazil [27,28], but also globally [26].

Not surprisingly, habitat losses among the BEs located in the Central region of the Cerrado, and the northernmost and southernmost BEs (Fig 2) were not significantly different, possibly a consequence of the south-northern [27,28] contiguous pattern of deforestation in the Brazilian Cerrado. This gradient is in consonance with the recent extensive occupation of Central Brazil [27], which has suffered important losses in habitat cover in the past 40 years, due to the expansion of agribusiness for exportation [25,27,28], and to the reallocation of the Country capital from Rio de Janeiro to Brasília in the early 60’s, coupled with an expansion of the road network and an intensive urbanization of Central Brazil [83]. Furthermore, future projections of habitat loss in the Cerrado point towards a maintenance of the current expansion rate of agriculture [84]. The Cerrado will continue to be the main region for landscape conversion in Brazil [85], and, proportionally, BEs in the Southern region of the Cerrado will probably continue to be heavily affected by deforestation (only 34% of the original cover remains). It is clear that the Cerrado could be part of the ongoing worldwide process of species loss due to anthropic causes [86].

When comparing priority areas either considering or neglecting habitat loss, we found different regions for implementing conservation efforts (Fig 3B and 3D). Facing the current picture of habitat loss of the Cerrado, the best 10–25% priority areas for conservation areas appeared in central and northern parts of the Cerrado (Fig 3C and 3D). This situation is possibly a consequence of the loss of important areas for conservation in the southern portion of the Cerrado (Fig 1B and 1C) a result that we also obtained for the losses within each BE (Fig 2C). This pattern is also perceived with little modifications for priority areas in the northern portion of the Cerrado, and the, comparatively, small losses of this region (Fig 2A and 2B). Central and northern portions of the Cerrado have only recently been occupied, and their biogeographical heritage is thus less impacted by habitat loss, and more prone to be represented by larger reserves. This is a typical situation in long-settled regions of the world: by the time biodiversity conservation became a social priority, only a non-random subset of the original habitat types was available for conservation management [87]. This biased distribution of conservation units, and of distribution of suitable habitats for conservation initiatives, is found in most terrestrial ecosystems and regions [80,88,89].

### Where and why to protect

Historical factors are important to the formation of Squamate faunas [16,38,90], and assuming that species forming BEs are likely to share a common biogeographic history [17], our data shows that we may be losing historical information in the Cerrado. As in the Cerrado the first areas to be loss are exactly the interfluvial plateaus [28], key biogeographical areas patterns for Squamates [16], and for multiple vertebrate groups in the Cerrado [15,29].This scenario is a consequence of the non random deforestation pattern in the Cerrado due to the expansion of mechanized agriculture [27,28], since, in general, the best farming land are tabletop savannas, these are the first areas to be cleared [87].

Moreover, our results indicate that the current PA system in the Cerrado is not representative of regional biogeographic regions and does not take into account ancient and current patterns of diversity distribution [2]. Hence, highly important regions for the conservation of biogeographical patterns in the Cerrado are both negatively impacted by deforestation and poorly protected (Fig 3). This indicates that decisions about the location of PA were opportunistic, based more on the availability of an area for conservation management, scenic beauty and recreational values, regardless of underlying biogeographical and biodiversity patterns (see discussions in [91–93]). Also, the general lack of protection in agriculture prone regions may stem from an opportunistic reserve selection, favoring globally the protection of dry, unfertile, rocky or steep habitats [92, 94],.

As the Cerrado rapidly disappears [28], we point out that it is crucial to both expand the Cerrado PA network in western Cerrado, mainly in priority areas VI (Chapada dos Guimarães) and VIII (Serra da Bodoquena), through a systematic approach (sensu [45]) and to maintain and properly manage the small remnants and PA in the southern Cerrado [80,95], priority areas I (São Paulo) and VII (Três Lagoas), in order to effectively protect biogeographical and evolutionary information on the richest and most imperiled savanna region in the planet.

## Supporting Information

S1 Fig Regions for Cerrado squamate conservation. Suitable regions for implementing conservation actions for Cerrado endemic Squamates, considering both current habitat loss and protected area coverage. Gradient colors are as follows: dark red, the best 2% of the landscape for conservation efforts; red, the best 2–5%; orange, the best 5–10%; dark yellow, the best 10–25%; yellow, 25–50%; pale yellow the remaining 50–100%. Cerrado original cover is represented in light gray. Protected areas (IUCN categories I-IV) are represented in green.
(TIF)

S1 Table Distribution and Threat data for all 49 endemic squamates forming Cerrado Biotic elements. Group: amp—amphisbaenians; liz—lizards; ser—serpentes. Taxon: species names according to Bérnils & Costa (2012). NR: number of locality records for each species. PAC: Protected Area Coverage, calculated by the sum of the species original distribution covered by protected areas (IUCN categories I-IV). % PA: Percentage natural vegetation within range covered by protected areas. BE: Endemic Biotic Element number as in Nogueira et al. (2011). OR: Expected area of natural vegetation within range in the original Cerrado coverage (in km²). 2010: Expected area of natural vegetation within range in the year 2010 (in km²). BE 2010: Expected area of natural vegetation within range in the year 2010 if losses were homogeneous throughout the BE (in km²).
(XLSX)

S2 Table Area loss comparison among Biotic Elements. Legend: BE: Biotic Element numbered and named as in Nogueira et al. (2011). Denomination: Biotic element's denomination as in Nogueira et al. (2011). D: Kolmogorov-Smirnov test result. P-value: For the statistical analyses we considered a significance level of 0.05.
(XLSX)
