# Supplementary figures and images for: Conserving Biogeography: Habitat Loss and Vicariant Patterns in Endemic Squamates of the Cerrado Hotspot
Source: PLoS One. 2015 Aug 7;10(8):e0133995. doi: 10.1371/journal.pone.0133995 (PMC4529144; doi:10.1371/journal.pone.0133995)

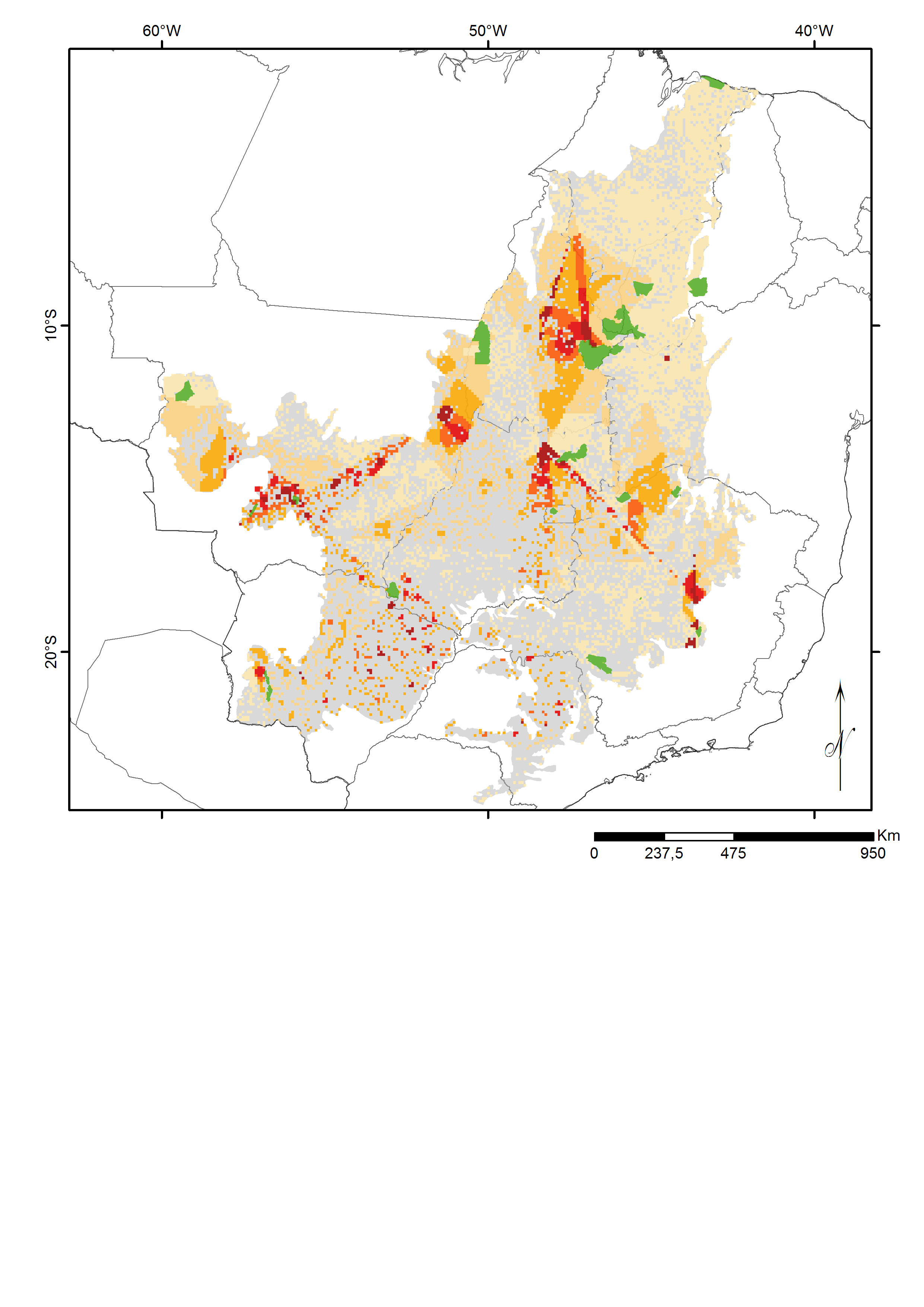

Supplement: S1 Fig — Suitable regions for implementing conservation actions for Cerrado endemic Squamates, considering both current habitat loss and protected area coverage. Gradient colors are as follows: dark red, the best 2% of the landscape for conservation efforts; red, the best 2–5%; orange, the best 5–10%; dark yellow, the best 10–25%; yellow, 25–50%; pale yellow the remaining 50–100%. Cerrado original cover is represented in light gray. Protected areas (IUCN categories I-IV) are represented in green. (TIF) [file pone.0133995.s001.tif]
